# A rare case of anterior inferior iliac spine osteochondroma causing external snapping hip syndrome: a case report

**DOI:** 10.1097/RC9.0000000000000877

**Published:** 2026-08-25

**Authors:** Sileshi Serebe Zeleke, Getaneh Workneh Kassa, Demis Hailu Ashagrie, Wuletaw Muche Mihret, Demeke Yilkal Fentie, Gashaw Tigabu Mamo

**Affiliations:** a Department of Orthopedics, University of Gondar, College of Medicine and Health Sciences, Gondar, Ethiopia; b Department of Anesthesia, University of Gondar, College of Medicine and Health Sciences, Gondar, Ethiopia; c Department of Pathology, University of Gondar, College of Medicine and Health Sciences, Gondar, Ethiopia

**Keywords:** anterior inferior iliac spine, case report, osteochondroma, pelvic tumor, snapping hip

## Abstract

**Introduction and importance:**

Osteochondroma is the most common benign bone tumor; however, involvement of the anterior inferior iliac spine (AIIS) is rare. Such lesions may lead to mechanical symptoms due to impingement on surrounding soft tissues, resulting in unusual presentations such as snapping hip syndrome.

**Presentation of the case:**

A 20-year-old male presented with a 3-year history of painful external snapping over the left hip, aggravated by movement. Physical examination revealed a firm, non-tender swelling in the left anterior groin region. Radiographs and CT imaging demonstrated a sessile bony lesion arising from the left AIIS, consistent with osteochondroma. The lesion was excised via an anterior Smith–Petersen approach. Histopathology confirmed osteochondroma. Postoperatively, the patient had complete resolution of symptoms and returned to full activity without recurrence at 1-year follow-up.

**Clinical discussion:**

An AIIS osteochondroma is an extremely rare cause of external snapping hip syndrome. The condition results from mechanical irritation of the rectus femoris due to a bony prominence at its origin. CT imaging is essential for diagnosis, while complete surgical excision remains curative with excellent functional outcomes.

**Conclusion:**

An AIIS osteochondroma should be considered in the differential diagnosis of snapping hip syndrome. Early recognition and surgical excision result in complete symptom resolution and a low risk of recurrence.

## Methods

This case report is conducted following the SCARE criteria for surgical case reports^[^1^]^.

## Introduction

Osteochondroma, also known as an osseocartilaginous exostosis, is the most common benign bone tumor, accounting for approximately 20–50% of benign bone tumors and 10–15% of all bone tumors^[^1^]^. These lesions typically develop in the metaphyseal regions of long bones such as the femur, tibia, and humerus^[^2^]^. Pelvic osteochondromas are rare, representing less than 5% of all cases^[^3^]^. When located at the anterior inferior iliac spine (AIIS), they may impinge on nearby soft tissues, particularly the rectus femoris or iliotibial band, resulting in external-snapping hip syndrome – a mechanical phenomenon characterized by an audible or palpable snapping sensation during hip movement^[^4^]^. We report a rare case of AIIS osteochondroma causing external snapping hip in a young adult, successfully managed by surgical excision.

HIGHLIGHTS

- Osteochondroma is the most common benign bone tumor but is rarely found in the pelvic bones.
- Osteochondroma should be considered as a differential diagnosis in external snapping hip syndrome.
- Complete resection of the tumor is the treatment of choice.
- The prognosis is excellent, with no recurrence and complete resolution of symptoms.

## Presentation of the case

**Figure 1.**
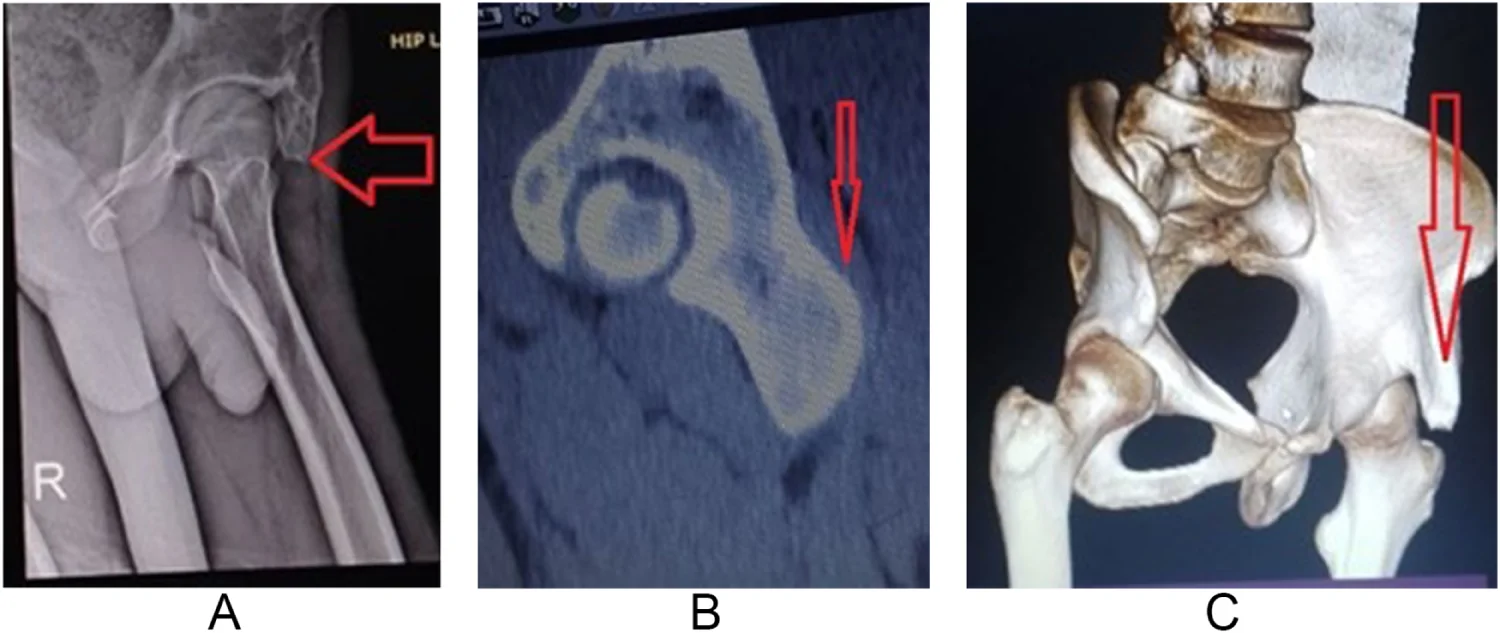
(A) Plain radiograph (anteroposterior view) showing bony exostosis (arrow) from the anterior aspect of the iliac bone. The lesion demonstrates continuity with the underlying cortex. (B, C) CT scans showing a sessile osteochondroma arising from the left anterior inferior iliac spine. The lesions (arrows) show continuity of cortical and medullary bone with the native iliac bone, characteristic of an osteochondroma.

A 20-year-old male college student, with a body mass index of 19.7 kg/m^2^, presented with a 3-year history of a painful snapping sensation over the left hip. He had no known previous medical or surgical illnesses, no known drug allergies, and no family history of a similar illness. The symptoms were aggravated by walking, running, or flexion-extension of the hip and were associated with mild discomfort, but no neurological symptoms. Physical examination revealed a firm, non-tender, immobile swelling in the left anterior groin region, clinically palpable closer to the anterior superior iliac spine, while imaging demonstrated that the lesion originated from the underlying AIIS. The snapping was reproduced during active hip flexion and extension, and it resolved at rest. There was no limb-length discrepancy or neurovascular deficit. Plain radiograph (Fig. 1A) showed a bony outgrowth arising from the anterior aspect of the left iliac bone. A CT scan (Fig. 1B and C) demonstrated a sessile, corticated bony lesion continuous with the cortex and medullary cavity of the left AIIS, diagnostic of osteochondroma.

**Figure 2.**
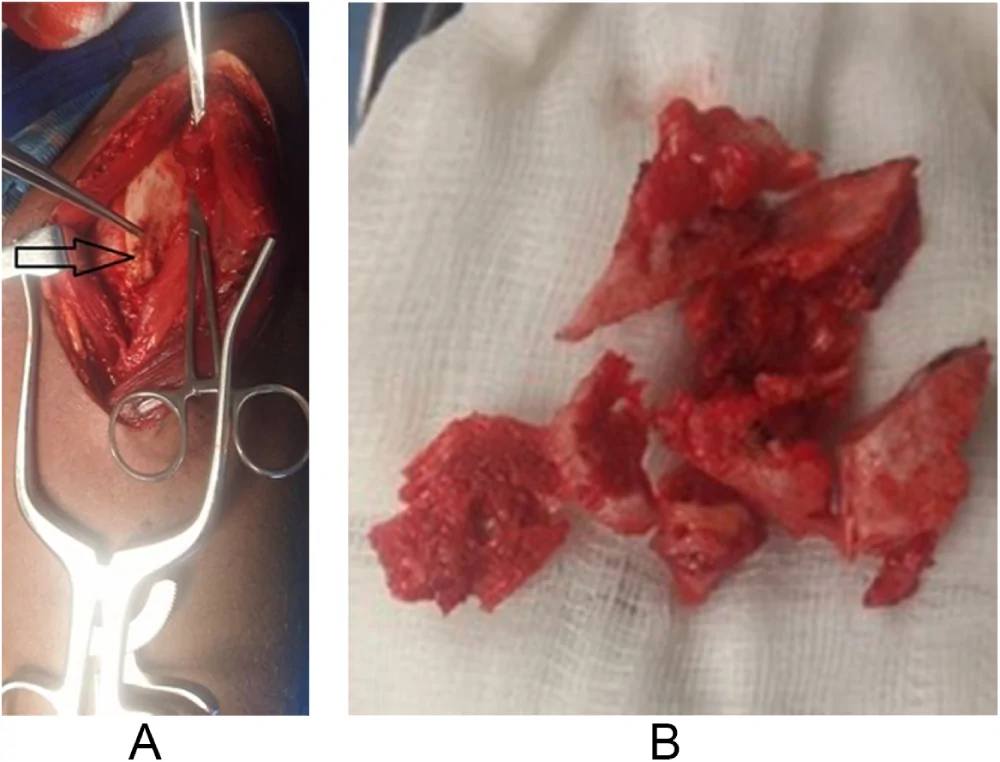
(A, B) Intraoperative images of a bony exostosis arising from the AIIS and an excised specimen showing irregular cartilaginous and bony fragments.

Under spinal anesthesia, the patient was positioned supine and underwent excision of the lesion through an anterior Smith–Petersen approach. The lateral femoral cutaneous nerve was identified and protected throughout the procedure. The tensor fasciae latae and rectus femoris were carefully retracted, revealing a well-circumscribed bony projection arising from the AIIS. The lesion was excised flush with the native bone (Fig. 2A and B). The operative time was approximately 1 hour, and the estimated blood loss was 100 mL. Postoperatively, the patient remained stable and was discharged on the second postoperative day. Early ambulation was encouraged, and no weight-bearing restrictions were imposed. The wound healed uneventfully without complications. The wound healed well without complications. The snapping sensation resolved completely. At the 1-year follow-up, the patient remained asymptomatic, with full range of motion and no recurrence on clinical and radiologic evaluation. The clinical timeline is summarized in Table 1.

**Table 1:** Clinical timeline frame.

Time point	Event
3 years before presentation	Onset of painful sensation over left hip
Day of presentation	Clinical evaluation and examination performed
Day 1	Plain radiograph and CT scan done, AIIS Osteochondroma considered
Day 2	Surgical incision via anterior Smith-Peterson approach under spinal anesthesia
Day 4	Discharged from hospital; early ambulation encouraged; full weight bearing as tolerated
2 weeks postoperatively	Histopathologic examination confirmed Osteochondroma
6 weeks postoperatively	Complete resolution of snapping; full range of motion
1 year postoperatively	Asymptomatic; no recurrence on clinical and radiologic evaluation

**Figure 3.**
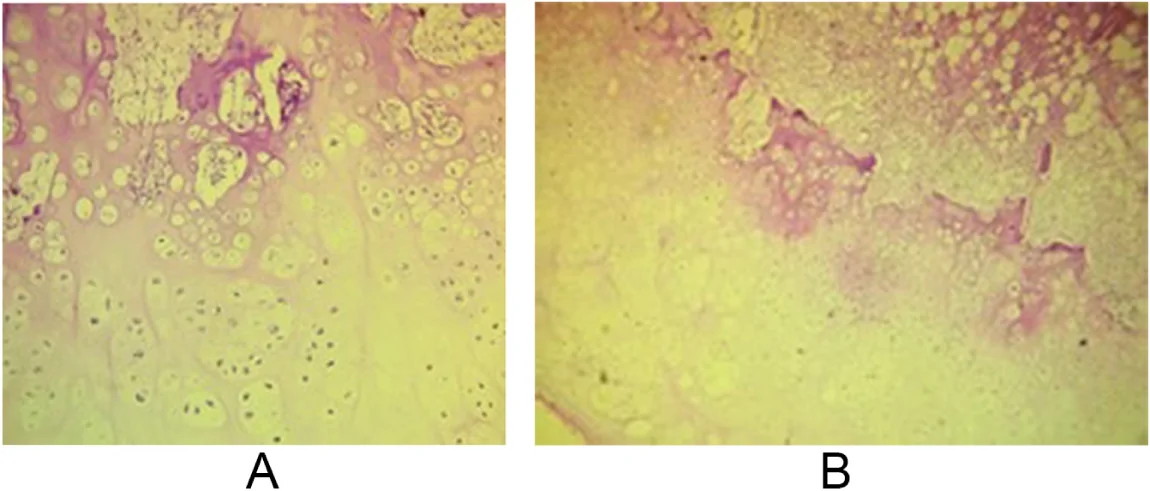
(A, B) Histopathologic examination showing cartilaginous tissue with chondrocytes arranged in lacunar spaces, consistent with osteochondroma.

The diagnosis of osteochondroma was made based on characteristic radiological and intraoperative findings demonstrating continuity of the cortical and medullary bone with the native AIIS, consistent with a benign osteochondroma. Histopathological evaluation demonstrated a bone tumor with a cartilaginous cap containing chondrocytes within lacunar spaces, consistent with osteochondroma, and the lesion was completely excised (Fig. 3A and B).

## Discussion

Osteochondromas are developmental lesions arising from aberrant cartilage growth at the growth plate^[^5^]^. While they commonly occur in long bones, pelvic osteochondromas – particularly those involving the AIIS – are exceedingly rare^[^6^]^. The AIIS serves as the origin of the direct head of the rectus femoris muscle, and a bony prominence in this region may mechanically irritate or displace nearby tendons, producing external snapping hip syndrome^[^7^]^. Most osteochondromas are asymptomatic and are often discovered incidentally; when symptomatic, clinical features are usually related to mechanical irritation or impingement, while complications such as malignant transformation are rare^[^8^]^.

Snapping hip syndrome is broadly classified into internal, external, and intra-articular types. External snapping is typically attributed to the iliotibial band or gluteus maximus moving over the greater trochanter, whereas internal snapping is related to iliopsoas tendon movement. In contrast, the present case represents an atypical extra-articular cause of snapping hip due to a bony prominence at the AIIS. The osteochondroma likely altered the normal gliding mechanism of the rectus femoris tendon, producing a reproducible snapping phenomenon during hip flexion and extension.

Differential diagnoses considered included myositis ossificans, parosteal osteosarcoma, subperiosteal hematoma, and chondrosarcoma. Myositis ossificans was ruled out by the absence of a history of trauma and the lack of a characteristic zoning pattern on imaging. Parosteal osteosarcoma was excluded due to the absence of aggressive features such as cortical destruction or soft tissue extension, and the presence of corticomedullary continuity with the native bone. Subperiosteal hematoma was considered unlikely given the absence of trauma history and the lesion’s bony nature. Chondrosarcoma was ruled out based on the patient’s young age, lack of aggressive radiographic features, and histopathological confirmation of a benign cartilaginous cap without atypical cells. CT is especially useful in defining the lesion’s cortical and medullary continuity, guiding safe and complete resection while minimizing neurovascular risk^[^9^]^.

The literature contains very few reports of AIIS osteochondroma causing snapping hip, all managed successfully with excision^[^6,7^]^. Another case reported in the literature described a similar presentation in a young female patient with AIIS osteochondroma causing snapping hip and was successfully treated with complete excision, with excellent functional outcome^[^4^]^. In all reported cases, including ours, patients presented with painful mechanical snapping aggravated by hip movement, with imaging confirming a sessile bony lesion arising from the AIIS with characteristic corticomedullary continuity. Surgical excision via an anterior approach resulted in complete symptom resolution in all cases, with no recurrence reported at follow-up^[^4,6,7^]^. The consistency of these outcomes across reported cases supports the conclusion that complete surgical excision is curative for AIIS osteochondroma presenting as snapping hip syndrome. Although the condition is rare, these cases highlight the importance of considering osteochondroma in the differential diagnosis of young patients with mechanical snapping hip unresponsive to conservative management.

Our patient’s rapid postoperative recovery and symptom resolution are consistent with these outcomes, supporting the conclusion that early surgical management provides curative results. Complete excision, including removal of the cartilage cap, prevents recurrence^[^10^]^. Table 2 summarizes previously reported cases of AIIS osteochondroma causing snapping hip syndrome.

**Table 2:** Summary of reported similar cases.

Reference	Age/sex	Presentation	Imaging	Treatment	Outcome
8	25/M	Painful snapping hip	CT/MRI	Surgical excision	Symptom free at 1 year
5	30/F	Painful snapping hip	CT	Surgical excision	Excellent; no recurrence
7	Various	Pelvic osteochondroma	Radiograph/CT	Surgical excision	Good functional recovery
Present case	20/M	Painful snapping hip	Radiograph/CT	Surgical excision	Symptom free at 1 year; no recurrence

Functional recovery is typically rapid, with full motion regained within a few weeks postoperatively, as seen in our case. The absence of recurrence at 1-year follow-up supports the benign and non-recurrent nature of these lesions when excised completely. The patient reported significant improvement in his quality-of-life following surgery. He stated that the snapping sensation and associated pain had completely resolved, allowing him to return to his daily activities, including walking and running, without discomfort. He expressed satisfaction with the surgical outcome and the recovery process.

## Conclusion

An AIIS osteochondroma is a rare cause of external snapping hip syndrome. Clinicians should consider this diagnosis in young patients presenting with mechanical snapping that is unresponsive to conservative management. Surgical excision is curative, with excellent functional outcomes and a minimal risk of recurrence.

Key take-home messages include: (1) AIIS osteochondroma should be included in the differential diagnosis of snapping hip syndrome, especially in young patients; (2) CT imaging is essential for accurate diagnosis and preoperative planning; and (3) complete surgical excision provides excellent outcomes with a low recurrence risk.

Strengths of this report include a comprehensive clinical, radiological, and histopathological correlation with a 1-year follow-up. Limitations include a single-case report design and the absence of a validated hip outcome score.

## Data Availability

The authors of this manuscript are willing to provide any additional information regarding the case report.
